# Effect of Maternal Preeclampsia on Hematological Profile of Newborns in Qatar

**DOI:** 10.1155/2020/7953289

**Published:** 2020-03-14

**Authors:** Mohammad A. A. Bayoumi, Abir A. H. Ali, Sara G. Hamad, Alaa A. M. Ali, Einas E. Elmalik, Mohamed M. I. R. Elkalaf, Bassem A. A. Moustafa, Deena A. D. A. Shaltout, Prem Chandra, Lisa J. Langtree, Noimot O. Olayiwola

**Affiliations:** ^1^Neonatal Intensive Care Unit (NICU), Women's Wellness and Research Center (WWRC), Hamad Medical Corporation (HMC), P.O. Box 3050, Doha, Qatar; ^2^Department of Medical Education, Hamad Medical Corporation (HMC), P.O. Box 3050, Doha, Qatar; ^3^Medical Research Center, Hamad Medical Corporation (HMC), P.O. Box 3050, Doha, Qatar; ^4^Medical Records Department, Women's Wellness and Research Center (WWRC), Hamad Medical Corporation (HMC), P.O. Box 3050, Doha, Qatar; ^5^Corporate Communication Department, Hamad Medical Corporation (HMC), P.O. Box 3050, Doha, Qatar

## Abstract

**Background:**

Preeclampsia is a major cause of pregnancy-related maternal, fetal, and neonatal morbidities and mortalities. We aimed to review the effect of maternal preeclampsia on the hematological profile of newborns in the Qatari population.

**Methods:**

In this case-control study, we reviewed data and complete blood count results of neonates born to Qatari women diagnosed of preeclampsia in 2017 in comparison with data of a control group. Statistical analysis was done using unpaired *t*-test, chi-square test, and logistic regression analysis.

**Results:**

A total of 108 neonates of women with preeclampsia and 103 neonates of healthy normotensive women were recruited. The mean weight, length, head circumference, placental weight, and gestational age were significantly lower (*P* < 0.05) in neonates born to women with preeclampsia. Only 13% of babies born to women with preeclampsia developed neonatal thrombocytopenia which is significantly higher compared to only 2% in the control group (chi-square *χ*^2^ = 9.14; *P* = 0.003). No significant difference (*P* > 0.05) was noted between the two groups regarding the white blood cells (WBC) or the absolute neutrophilic count (ANC). Multivariate logistic regression showed that the gestational age, birth weight, length, and ANC had significant association with preeclampsia (*P* < 0.05).

**Conclusions:**

We found that there was a positive association between preeclampsia and neonatal thrombocytopenia in the Qatari population. Prematurity, placenta insufficiency, fetal growth restriction, and need for neonatal resuscitation were significantly higher in babies born to women with preeclampsia. We recommend that hematological parameters of neonates of those women should be properly monitored to reduce the chances of developing complications.

## 1. Introduction

Hypertensive disorders are the most common medical problem during pregnancy. These disorders have been classified by the Working Group of the National High Blood Pressure Education Program (NHBPEP) (2000) into five types:

1. Gestational hypertension (formerly pregnancy-induced hypertension that included transient hypertension)
2. Preeclampsia
3. Eclampsia
4. Preeclampsia superimposed on chronic hypertension
5. Chronic hypertension

Preeclampsia toxemia (PET) is a major cause of pregnancy-related maternal morbidities. However, preeclampsia also carries higher rates of morbidities and mortalities in neonates [1, 2].

Many studies showed that neonates of hypertensive women, especially those who have preeclampsia, are more liable to have hematological permutation. Although the etiology and pathogenesis of preeclampsia is not fully known, a proinflammatory immune state prevails and can disrupt fetal hematopoiesis. Some of the effects on newborns include neonatal thrombocytopenia, neutropenia, a reduction in T regulatory cells, and an increased cytotoxic natural killer cell profile [3, 4]. There are no established international guidelines for routine screening of babies delivered to women with preeclampsia.

This study was aimed at reviewing the effect of maternal preeclampsia on the hematological profile of newborns as well as certain maternal and fetal outcomes and to compare it with those of healthy normotensive women in the Qatari population. Early neonatal hematological screening might help to decrease morbidity and improve growth, development, and survival of the baby.

## 2. Patients and Methods

A one-year case control study was conducted in the Neonatal Intensive Care Unit (NICU) of Women's Wellness and Research Center (WWRC) at Hamad Medical Corporation, Doha, Qatar. The study consisted of 108 Qatari neonates born to women with preeclampsia and 103 Qatari neonates born to healthy normotensive women. This study was approved by the appropriate ethics committee and has therefore been performed in accordance with the ethical standards laid down in the 1964 Declaration of Helsinki and its later amendments. It was approved by the Institutional Research Board (IRB) and was given the number MRC-01-17-148.

In 2017, we collected the data about the gestational age, growth parameters (weight, length, and head circumference), Apgar score, neonatal white blood cell count (WBC), absolute neutrophilic count (ANC), and platelet count (Plt) for each group.

The normal hematological values for this age group are shown in Tables 1 and 2.

### 2.1. The Inclusion Criteria

We considered babies born to Qatari women who suffered from preeclampsia and were admitted for delivery in Women's Wellness and Research Center in 2017.

We used the following criteria to diagnose preeclampsia [5]:

(1) Pregnancy older than 20 weeks' gestation
(2) Having hypertension
  - Systolic blood pressure (SBP) ≥ 140 mmHg or diastolic blood pressure (DBP) ≥ 90 mmHg on 2 occasions (at least 4 hrs. apart) in a previously normotensive patient
  - SBP ≥ 160 mmHg or DBP ≥ 110 mmHg on 1 occasion
(3) Having proteinuria
  - 24-hour urine: proteinuria ≥ 0.3 grams
  - Protein (mg/dL)/creatinine (mg/dL) ratio ≥ 0.3
  - Urine dipstick protein of 1+
(4) In a patient with new onset of hypertension without proteinuria, the new onset of any of the following is diagnostic of preeclampsia
  - Platelet count < 100,000/*μ*L
  - Serum creatinine level > 1.1 mg/dL or doubling of serum creatinine in the absence of other renal disease
  - Liver transaminase levels at least twice the normal concentrations
  - Pulmonary edema
  - Cerebral or visual symptoms

### 2.2. Exclusion Criteria

1. Babies with congenital malformations
2. Babies with severe birth asphyxia
3. Neonates of women with chronic illnesses
4. Maternal aspirin use

### 2.3. Statistical Analysis

- Data were coded and collected in an excel sheet
- No patients' identifiers were disclosed
- Descriptive statistics were used to summarize demographic, clinical characteristics, maternal and fetal outcome measures, and other related characteristics of the participants
- The normally distributed data and results were reported with mean and standard deviation (SD); the remaining results were reported with median and interquartile range (IQR)
- Categorical data were summarized using frequencies and percentages
- Associations between two or more qualitative variables were assessed using chi-square (*χ*^2^) test or Fisher's exact test as appropriate
- Quantitative data between two independent groups (preeclampsia vs. nonpreeclampsia) were analyzed using unpaired *t*-test or Mann–Whitney *U* test as appropriate depending on normality of the data distribution
- Logistic regression method was applied to determine and assess the effect of maternal preeclampsia on both maternal and fetal outcome measures (predictive values of each predictor)
- For multivariate logistic regression models, variables were considered if statistical *P* < 0.10 level in univariate analyses or if determined to be clinically important
- A two-sided *P* value < 0.05 was considered statistically significant
- All statistical analyses were performed using the IBM SPSS® Statistics for Windows, Version 22.0 (IBM Corp, Armonk, NY, USA) and Epi Info™ 2000 (Centers for Disease Control and Prevention, Atlanta, GA, USA)

## 3. Results

A total of 211 Qatari neonates were recruited, comprising 108 neonates of women with preeclampsia (cases) and 103 neonates of healthy normotensive women (controls). All the neonates in both categories (cases and control) were booked (they were registered cases who were on regular checkup). Details of study and control group are shown in Table 3.

In our study, we found that the mean weight, length, head circumference, placental weight, and gestational age were significantly lower in neonates born to women with preeclampsia than those of healthy normotensive women. The platelet counts of neonates born to women with preeclampsia were significantly lower than those of healthy normotensive women. Of note, no statistically significant difference was noted between the two groups in the white blood cells or the absolute neutrophilic count.

The results of logistic regression analysis testing for each potential predictor and its association with preeclampsia are presented in Tables 4 and 5. The results were presented with odds ratio (OR) and associated 95% confidence interval (CI). Logistic regression analysis revealed that placental weight, gestational age, baby's birth weight, length, head circumference, Apgar score at 5 minutes, and baby's platelet count were significant potential predictors associated with an increased risk for preeclampsia (*P* < 0.001), whereas baby's ANC and WBCs and mother's age did not show any significant association with preeclampsia (*P* > 0.05). Using multivariable logistic regression analysis controlling and adjusting for all other potential covariates and predictors considered in the univariate analysis as presented in Table 4, gestational age, baby's birth weight, length, and ANC showed strong and significant association with preeclampsia (Table 5, multivariate logistic regression).

## 4. Discussion

Hypertensive disorders are the most common medical complications in pregnancy and major causes of maternal, fetal, and neonatal morbidity and mortality. Preeclampsia is a great challenge to obstetricians because its cause is unknown, its pathophysiology is complex and incompletely understood, its diagnosis may be difficult to determine, there are no effective treatments, and antenatal care involves a difficult balance between the risks for women to continue pregnancy and those for the baby's early birth.

Newborn infants exposed to preeclampsia in utero are prone to short-term morbidity such as respiratory distress syndrome, bronchopulmonary dysplasia, and gastrointestinal problems. These problems could be related not only to preeclampsia itself but also to prematurity or fetal growth restriction (FGR), which frequently occurs in babies exposed to preeclampsia in utero.

This study was conducted in accordance with prevailing ethical principles and reviewed by an Institutional Review Board.

The data from this study shows significantly lower gestational age, placental weight, birth weight, length, and head circumference in babies born to women with preeclampsia in comparison with those who were born to healthy normotensive women. Low Apgar score both at 1 and 5 minutes was significantly more frequent in babies of women with preeclampsia than those of normotensive women.

Certain hematological derangements occur in newborns exposed to this disorder, especially thrombocytopenia and neutropenia. Neonatal thrombocytopenia is typically defined as a platelet count of less than 150,000/*μ*L. Degrees of thrombocytopenia can be further subdivided into mild (platelet count 100,000 to 150,000/*μ*L), moderate (50,000 to 99,000/*μ*L), and severe (<50,000/*μ*L).

Most of the cases of neonatal thrombocytopenia secondary to maternal preeclampsia are not manifested clinically; however, a small percentage might manifest with severe neonatal thrombocytopenia typically defined as a platelet count less than 50,000/*μ*L or by overt bleeding. The pathogenesis of thrombocytopenia among infants born to women with preeclampsia is unknown [6]. It might be related to exposure to intrauterine hypoxia with subsequent suppression of the megakaryocytic proliferation.

Neonatal neutropenia (defined as absolute neutrophil count of less than 500) secondary to maternal preeclampsia has no fully elucidated mechanism. One potential mechanism is that preeclampsia, and the resultant uteroplacenta insufficiency, inhibits fetal bone marrow production of the myeloid lineage manifested by a decrease in neutrophil production [7, 8].

In our study which included only Qatari babies delivered to women with preeclampsia, 13% of the babies developed neonatal thrombocytopenia, which is significantly lower when compared with 22% found in a similar study by Sivakumar et al. However, this 13% is a significantly higher percentage than our findings in the control group, which is only 2%. Other studies showed variable incidences of thrombocytopenia as 33% by Harm et al. [9], 34% by Eltink et al. [10], and 36% by Bhat and Cherian [11]. This variation might be related to the obstetric attempts made to improve assessment and surveillance of women who have had preeclampsia as well as the socioeconomic standard of the population.

Another highlighting result in our study is that leukopenia was present only in 11% of cases and 6% of the controls. We did not find any statistically significant difference between cases and controls in the white blood cell (WBC) count and the absolute neutrophilic count (ANC). Similarly, Sivakumar et al. also reported no significant change in neutrophil count. On the other hand, Bolat A et al. reported that the total count of lymphocyte, eosinophil, monocyte, and neutrophil was significantly lower in babies of women with preeclampsia in comparison to babies born to healthy normotensive women [12]. Meher et al. documented only change in neutrophil count [13]. Harms et al. demonstrated leukopenia in 21% of the affected infants [9].

Multivariable logistic regression analysis showed that the gestational age, birth weight, length, and ANCs had significant association with preeclampsia. We did not find in the literatures a similar study with logistic regression analysis to compare its findings with our study.

Despite the relatively low incidence of neonatal thrombocytopenia and neutropenia in our study population which might be related to the quality of the antenatal care and socioeconomic standard of the population, preeclampsia continues to be a common and serious complication of pregnancy and a cause of great concern because of the high rates of adverse maternal, fetal, and neonatal outcomes.

We believe that a multidisciplinary, collaborative approach between the fields of maternal-fetal medicine and neonatology is necessary to weigh the maternal and fetal risks of prolonging the pregnancy versus the potential benefits of further fetal maturation across most gestational ages.

Further prospective studies with higher sample size to understand the pathogenesis of neonatal thrombocytopenia because of maternal preeclampsia are recommended. A better understanding of the pathophysiology of the disorder may allow us to develop routine screening measures and strategies to prevent morbidities in those babies from birth throughout their adult life.

## 5. Conclusion

In our study, there was a positive association between preeclampsia and neonatal thrombocytopenia in the Qatari population. Prematurity, placenta insufficiency, fetal growth restriction, and need for neonatal resuscitation were found to be significantly higher in babies born to women with preeclampsia when compared with those who were born to healthy normotensive women. Hematological parameters of neonates of women with preeclampsia should be properly evaluated and monitored to reduce the chances of developing complications associated with these abnormalities, especially neonatal thrombocytopenia.

## Figures and Tables

**Table 1:** Normal hematologic values during the first two weeks of life in the term infants.

Value	Cord blood	Day 1	Day 3	Day 7	Day 14
Hemoglobin (gm/100 mL)	16.3	18.4	17.8	17.0	16.8
Hematocrit (%)	53.0	58.0	55.0	54.0	52.0
Red blood cells (cu. mm × 10^6^)	5.25	5.8	5.6	5.2	5.1
Mean corpuscular volume (m3)	107	108	99.0	98.0	96.0
Mean corpuscular hemoglobin (yy)	34	35	33	32.5	31.5
Mean corpuscular hemoglobin concentration (%)	31.7	32.5	33	33	33
Reticulocytes (%)	3-7	3-7	1-3	0-1	0-1
Nucleated red blood cells (cu. mm)	500	200	0-5	0	0
Platelets (1000/s/cu. mm)	290	192	213	248	252

**Table 2:** The white blood cell count and the differential count during the first two weeks of life.

Age	Leukocytes	Neutrophils			Eosinophils	Basophils	Lymphocytes	Monocytes
		Total	Segmented	Band				
Birth								
Mean	18.0	11000	9400	1600	400	100	5500	1050
Range	9.0-30.0	6.0-26			20-850	0-640	2.0-11.0	0.4-3.1
Mean %	—	61	52	9	2.2	0.6	31	5.8
7 days								
Mean	12200	5500	4700	830	500	50	5000	1100
Range	5.0-21.0	1.5-10.0			70-1100	0-250	2.0-17.0	0.3-2.7
Mean %	—	45	39	6	4.1	0.4	41	9.1
14 days								
Mean	11400	4500	3900	630	350	50	5500	1000
Range	5.0-20.0	1.0-5.0			70-1000	0-230	2.0-17.0	0.2-2.4
Mean %	—	40	34	5.5	3.1	0.4	48	8.8

**Table 3:** Comparison of maternal, fetal, and CBC parameters between PET cases and control cases.

Variable	Cases	Controls	*P* value
Mother's age (years)	31.28 ± 6.91	30.64 ± 5.72	0.246
Placental weight (gm)	593.43 ± 166.71	666.87 ± 103.81	<0.0001
Weight (gm)	2592.99 ± 792.65	3203.86 ± 457.10	<0.0001
Length (cm)	47.08 ± 4.99	50.29 ± 2.01	<0.0001
Head circumference (cm)	32.53 ± 3.04	34.23 ± 1.37	<0.0001
Gestational age (weeks)	36.78 ± 3.01	39.08 ± 1.21	<0.0001
Apgar score at 1 minute	8.52 ± 1.28	8.96 ± 0.36	<0.0001
Apgar score at 5 minutes	9.77 ± 1.07	9.99 ± 0.11	0.002
Baby's WBCs	11.32 ± 5.13	12.07 ± 3.88	0.232
Baby's absolute neutrophilic count (ANC)	5.16 ± 3.62	5.55 ± 3.82	0.444
Baby's platelet count	225.75 ± 86.27	301.37 ± 89.91	<0.0001

**Table 4:** Predictors associated with preeclampsia: univariate logistic regression analysis.

Predictors	Unadjusted odds ratio (OR)	95% CI for OR	*P* value
Mother's age (years)	1.02	0.99, 1.04	0.220
Placental weight			
>450 gm	1.0		
≤450 gm	16.99	6.59, 43.84	<0.0001
Birth weight			
>2500 gm	1.0		
≤2500 gm	12.35	7.45, 20.49	<0.0001
Length			
>45 cm	1.0		
≤45 cm	26.70	11.33, 62.91	<0.0001
Head circumference			
>31 cm	1.0		
≤31 cm	25.70	10.90, 60.62	<0.0001
Gestational age			
>37 weeks	1.0		
≤37 weeks	10.17	6.56, 15.78	<0.0001
Apgar score at 5 minutes	0.26	0.14, 0.48	<0.0001
Baby's WBCs	0.96	0.91, 1.02	0.236
Baby's absolute neutrophilic count (ANC)	0.97	0.90, 1.04	0.443
Baby's platelet count			
>150	1.0		
≤150	7.52	1.67, 33.98	0.009

CI: confidence interval; outcome variable: nonpreeclampsia (control group) was considered the reference group.

**Table 5:** Predictors associated with preeclampsia: multivariate logistic regression analysis.

Predictors	Unadjusted odds ratio (OR)	95% CI for OR	*P* value
Gestational age			
>37 weeks	1.0		
≤37 weeks	3.96	1.55, 10.12	0.004
Birth weight			
>2500 gm	1.0		
≤2500 gm	4.70	1.39, 15.86	0.013
Length			
>45 cm	1.0		
≤45 cm	6.58	1.15, 37.63	0.034
Baby's absolute neutrophilic count (ANC)	1.10	1.01, 1.20	0.043

CI: confidence interval; outcome variable: nonpreeclampsia (control group) was considered the reference group.

## Data Availability

The data that support the findings of this study are available upon reasonable request from the corresponding author. Data requests should be made to Bayoumi M., moh.abdelwahab@hotmail.com.

## Additional Points

*What is already known on this subject*? Preeclampsia has been established to impact the growth parameters of the newborns. Certain hematological derangements occur in newborns exposed to this disorder, especially thrombocytopenia and neutropenia. *What this study adds*? High-quality antenatal care for established preeclampsia by proper multidisciplinary family-centered care substantially reduced the impact of preeclampsia on newborns. The socioeconomic standard of the population is inversely proportional to the potential complications of preeclampsia. A better understanding of the pathophysiology of the disorder will help in developing routine screening measures and strategies to prevent morbidities in those babies from fetal through adult life.
